# Experiments on the Application of Suprarenin in Obstetrics

**Published:** 1908-03

**Authors:** Maximilian Neu

**Affiliations:** Gynecological Clinic, University of Heidelberg


### Experiments on the Application of Suprarenin in Obstetrics

By MAXIMILIAN NEU

Gynecological Clinic, University of Heidelberg

Reprint from the *Gynekologische Rundschau*, No. 12

(Provisional Communication)

[Specially translated for the BUFFALO MEDICAL JOURNAL]

ALLOW me, gentlemen, to report briefly on some experiments which I have undertaken as to a rational application of suprarenin to our special work. We are not interested in this connection with the question of suprarenin in local and spinal anesthesia, but simply with the use of the suprarenal extract alone. Before I report the results which I obtained in this field, it is pertinent to preface my remarks with an explanation of the pharmacodynamics of suprarenin.

The active substance of suprarenal extract was first prepared by Takamine under the name of adrenalin. Various investigators (Pauly, Aldrich, von Furth and others), have labored in the direction of determining the chemical formula and Aldrich's formula, empirically determined, has been frequently verified. F. Stolz, has even succeeded very recently in obtaining suprarenin by synthesis. We are therefore actually in a position to make use of an absolutely artificial chemically pure substance, from which possibility manifest advantages must redound. At first I made use of suprarenin Höechst. but had in view the newer substances which had already been placed at my disposal and which pharmacologically were identical with the suprarenal extract supplied from the laboratory of Beyer of Vienna, and Filehne of Breslau, for future introduction into clinical medicine. Suprarenin shows full conformity in its action with adrenalin and has the advantage in stability and cheapness. We are interested here only with the action on the uterus.

The English physiologist E. A. Schæfer some years ago called attention to the action of suprarenal extract in exciting contractions in the pregnant and nonpregnant uterus, and its exquisite hemostyptic properties especially in uterine hemorrhages; these statements, however, attracting no attention. Its introduction into gynecology was very gradual, and chiefly in the combination of suprarenin with local anesthetics; thus B. Müller,

Steinschneider, Cramer, Peters, H. A. Freund. Flatau, for example, reported trial excision of the portis vaginalis, vaginoperineorrhaphy, and especially intrauterine sounding in menorrhagia. A leading domain in obstetrics in which hemostasis is of paramount importance has not yet been opened up. In consideration of the action of suprarenin on the uterus one must therefore wonder the more, as the drug is an almost ideal uterine hemostatic.

How does suprarenin act? Kurdinowski who has occupied himself with thorough experiments on this subject came to the conclusion that the uterine contractions are dependent on the notable vascular contractions set up by suprarenin, and that its action upon the uterus was nonspecific, being only a part of a general contraction of all the arterioles of the body. The question as to so striking an action of suprarenin on the uterus is of vital importance, should the product find rational application in therapeutic doses. E. Kehrer has recently pronounced against this comprehension of suprarenin, basing his opinions on his very interesting and important physiological and pharmacological investigations on the living and surviving internal genitals of animals. A perusal of the records of Kehrer leaves no doubt that the suprarenin acts with as much vigor on the smooth muscle of the uterus, as upon the muscle of the bloodvessels themselves. Our time is insufficient to go further in this connection into other experimental literature on this subject. I will therefore pass on to the results of my own work.

Kehrer was probably the first to adduce exact proofs that suprarenin acts upon the human uterus. He used preparations from a myomatous uterus and from a metritic uterus associated with pyosalpinx. He used dilutions of from 1:400,000 to 1:2 mgms. Slow, energetic spontaneous movements were hastened and regularised. A second dose caused slight increase in tonicity. Here begin my researches. I studied in a series of cases, the sensitiveness of living uterine tissue to the irritation of suprarenin. I applied the drug in minimal doses to the uterine muscle, both in the unchanged uterus in connection with vaginofixation and in uteri the seat of myomatous and metritic alteration in connection with total extirpation. Finally, I made use of uteri which had been removed by laparatomy and placed for a short time in physiological salt solution at body temperature. In this manner I obtained uteri in the most varied anatomical relations and conditions. In one there was a fetus 36 cm. long. Another showed senile involution, and so forth.

All preparations showed in more or less pronounced fashion the phenomena of contraction and vasoconstriction. A particularly noteworthy case must not pass unmentioned. Patient, Mrs. S., age 32, III para, amputation of portis, vaginal fixation, anterior colporrhaphy and colpoperineorrhaphy. Uteromuscular injections of suprarenin (in anterior wall of corpus; fundus, posterior wall) set up such intense contractions that the previously soft uterus, corresponding to that of a multipara, but otherwise unaltered anatomically, became as hard as a stone, and the whole organ made rigid, underwent a regular elevation, at the same time becoming completely white in color. I therefore reached the same conclusions as Kehrer—namely, that the existence of a muscular irritative action of suprarenin upon the uterus cannot be doubted. Self evidently there exists side by side the vasoconstrictor phenomenon which need not surprise us when we bear in mind the action of suprarenal extract. I may therefore express here the following comprehension of the point of attack of suprarenin on the uterus: the poison acts by irritating the uteromuscular plus the vasomuscular components. This being the case we have everything most desirable for a uterine hemostatic. Fr. Pick once set up the conditions for the successful action of a hemostatic as based on experimental studies. The agent must bring about vasoconstriction to such a degree that the simultaneous rise in bloodpressure must be compensated in respect to its influence on the rapidity of outflow. Already there are present suggestive findings in this direction which speak for the fact that the known exquisite action of suprarenin in raising the bloodpressure is not to be feared from the point of view of hemostasis. I, myself, have directed attention to this point. the other component, the uteromuscular action, appears significant in that it can influence the vasoconstrictor only favorably as regards hemostasis.

After these preliminary studies I thought to test these properties of suprarenin in the pregnant human uterus. We know not only that the gravid uterus is highly sensitive to irritation of all kinds, but Kurdinowski's beautiful experiments have taught us that the uterus is irritable to suprarenin, while Kehrer has shown that in animals (cats) the nongravid uterus failing to respond to the action of suprarenin, the same uteri when pregnant give positive results expressed by an increase of toxicity. I was able to confirm Kehrer's findings in the gravid uterus of a dog. Particular advantages may be expected in connection with operations on the gravid uterus. With this in view I made preliminary investigations on pregnant rabbits and dogs, which together justified the theoretical assumptions. Time does not suffice to quote detailed accounts of these experiments. However, the clinical case which follows should prove of interest.

The most important operation which we are in a position to carry out upon the pregnant uterus is Cesarean section. From this point of view, all possibly indicated preliminary investigations were made. The hemorrhage from Cesarean section, since this operation was first performed, is one of the most dreaded complications. That is shown by the number of measures which have been devised for hemostasis. Upon this account distinguished obstetricians have counseled the substitution of the Porro operation. But even the omission of the preventive elastic ligature, and the recently warmly recommended prophylactic injection of ergotin (Olshausen) can protect us from either primary or secondary atonic hemorrhage only with the slightest degree of certainty. Hence the operator cannot reckon upon anything more definite (bearing in mind the experience of evil results) than an "armed peace" in the shape of preparations for extirpating the uterus should hemorrhage require it. Naturally, it will always be difficult to determine when the "peace" must give way to intervention. There is a certain reproach connected with the conditions which demand a hasty resort to this method of hemostasis. I am far removed from taking a position in this very important matter. If one surveys the means at our disposal today for rational hemostasis in Cesarean section, it appears to me that any new attempt to enhance the security of this step cannot but be welcomed.

Case—On May 26th, 1907, at 3 o'clock A.M., a patient was brought to the clinic who had been in labor for twelve hours; up to four years previous she had been perfectly healthy. She then took sick with pains in the joints which became smaller; was pregnant at the time. The symptoms increased. Late in 1905 admitted to the medical clinic for osteomalacia, and was treated for nine months with phosphorised codliver oil. Five labors, all spontaneous, the last November 21, 1902. Examination of pelvis gave the following measurements: Di. Spin. 27; crist. 29; troch 28; ext. 17¼, tub. isch, 10½; conj. vera, about 5¼. Typhoid osteomalacic pelvis. First oblique position.

Upon examination by the resident obstetrician the waters broke. Patient before admission had been examined by a very uncleanly midwife. During clinical observation extremely violent pains were present, without any phenomena of dilatation. My chief ordered further exploration for the purpose of deciding upon the degree of narrowing of the pelvis; and as to the Cesarean section with eventual total extirpation of the uterus, bearing in mind the possibility of infection already under way. Removal of the adnexa was also indicated by reason of the osteomalacia.

At 4:30 P.M. operation. Chloroform narcosis. Transverse fundal Cesarean section and supravaginal amputation (removal of both adnexa) ; stumps treated retroperitoneally. Incision 15 cm. long, through the extremely thinned abdominal walls. Uterus rolled out. Suprarenin injected without delay 1:1000 (a hypodermic syringeful, was injected in 3 parts in as many places in the anterior wall of the uterus deeper by chance than corresponded to the chosen site of the incision. The uterus straightened up, became hard and pale. Peritoneal coat corresponding to injection zone became wrinkled. Transverse incision made a little below fundus. In making the incision the placental site was encountered, and here occurred the only hemorrhage. For the rest the loss of blood was minimal, although no bloodvessels were clamped. Placenta together with fetus in its envelopes removed. No escape of amniotic fluid. Provisional suture (continuous silk) of uterine incision. No hemorrhage from latter. Uterine wall firm; appeared completely anemic. Typical extirpation of uterus. Divided close above the os (instead of the vagina, the strongly drawn out, thinned segment of the cervix encountered). Supravaginal amputation ring cared for, retroperitoneal position of amputation stump. Smooth healing (first evening 38. 3°C; 6th day, 38°). Patient discharged cured on sixteenth post-operative day.

What may we conclude from this case? By means of minimal doses of suprarenin we are able to throw at once the parturient uterus into violent contractions, the entire organ being seen at the same time to be anemic. Therefore we may perform Cesarean section practically under complete absence of blood.

I conclude as a result of my experience with animal experiments, and the positive reproduction of these results in the human subject, that in suprarenal extract we have a highly efficacious remedy for the gravid uterus, the hemostatic property of which through its duplicity of action,—on the contractile elements of the uterus itself and its bloodvessels,—renders it deserving of consideration for the future bloodless operations on the pregnant and parturient uterus. The aim of my communication is to call attention to these points of superiority and to ask that they be tested by others.

